# Multi-Response Optimization of the Malting Process of an Italian Landrace of Rye (*Secale cereale* L.) Using Response Surface Methodology and Desirability Function Coupled with Genetic Algorithm

**DOI:** 10.3390/foods11223561

**Published:** 2022-11-09

**Authors:** Antonio Calvi, Giovanni Preiti, Marco Poiana, Ombretta Marconi, Martina Gastl, Martin Zarnkow

**Affiliations:** 1 Department of AGRARIA, University Mediterranea of Reggio Calabria, 89122 Reggio Calabria, Italy; 2 Italian Brewing Research Centre, University of Perugia, via San Costanzo s.n.c., 06126 Perugia, Italy; 3 Research Center Weihenstephan for Brewing and Food Quality, Technical University of Munich, Alte Akademie 3, 85354 Freising, Germany

**Keywords:** rye, landrace, response surface methodology, malting optimization, desirability function, genetic algorithm

## Abstract

Rye is used in some applications in the food and beverage industry and for the preparation of functional foods. It is an interesting raw material in malting and brewing due to its characteristic contribution to the beer’s color, turbidity, foam and aroma. The aim of this work was to optimize the micro-malting process of a rye landrace. The response surface methodology (RSM) was applied to study the influence of three malting parameters (germination time, germination temperature and degree of steeping) on the quality traits of malted rye. Long germination times at high temperatures resulted in an increase in the extract and Kolbach index. The model for the apparent attenuation limit showed a particular pattern, whereby time and temperature inversely influenced the response. The lowest viscosities were determined in the worts produced from highly modified malts. Optimization of the variables under study was achieved by means of a desirability function and a genetic algorithm. The two methodologies provided similar results. The best combination of parameters to optimize the malting process on the rye landrace under study was achieved at 6 days, 12 °C and 44 g/100 g.

## 1. Introduction

In recent years, the evolution and competition between beer multinational corporations and craft breweries have become increasingly intense [1]. Local companies rely on marketing strategies that emphasize the origin of raw materials and the link with the territory. Indeed, according to a study on the Italian craft beer sector, more than 70% of the surveyed breweries included information on the origin of the raw materials [2]. Such an approach is based on the concept of neolocalism, defined as “a conscious effort by businesses to foster a sense of place based on attributes of their community” [3]. Research is interested in the reintroduction of traditional rye varieties that enable high yields in marginal and poor soils [4]. Landraces are autochthonous and genetically related varieties that indeed exhibit stable yields in low-input environments over time [5]. The trend towards the rediscovery of local genetic resources can also be traced to the Calabria region (southern Italy). Rye landraces are typically cultivated in the mountainous areas of the region, above an altitude of 750 m. Interestingly, this rustic cereal is known by different names in the local dialects, e.g., ‘*iermunu*’ or ‘*granuiermanu*’, words that have a peculiar assonance with Germany, the country from which rye was allegedly originally imported [6]. Local craft breweries are strongly interested in locally grown raw materials and beers containing rye are part of their special releases. However, malts derived from local grains are essentially unavailable.

Rye (*Secale cereale* L.) is a species belonging to the *Poaceae* family. It is genetically close to wheat to the extent that the commercial hybrid Triticale (x *Triticosecale* Wittmack) is derived from them [7]. The domestication and spread of rye as a crop in human civilization are shrouded in mystery. Native to the Anatolian peninsula, it initially spread to central Europe as a weedy plant [8]. A variety of controversial theories have tried to shed light on the species. Anyhow, it is generally agreed that it was not among the first cereals cultivated at the dawn of agriculture [9]. Considered a minor cereal, it is second only to wheat for the production of bread and other bakery products [10]. Its cultivation is generally restricted to cold climates and harsh environments where other cereals would struggle to thrive [11]. Total world production in 2020 was 15,022,273 tons, with Germany, Poland and the Russian Federation among the top producers. From 2010 to 2020, Italy’s rye production was fluctuating with an average yield of 13,030.5 tons [12]. Rye has a carbohydrate content varying between 66% and 80%, while total protein ranges from 6.5 to 14.5% [13]. It represents an outstanding source of dietary fiber, bioactive compounds [14], polyphenols, flavonoids and thiols, whose contents are strongly affected by genotype [15]. Health benefits related to its consumption as a whole grain have been reported to improve gut function [16]. Secondary metabolites known as benzoxazinoids, which may exert antimicrobial and anticarcinogenic roles [17], have been detected in wheat- and rye-based beers [18]. Remains of unmalted rye were found at an archaeological site in Berlin, along with oats and germinated barley. Albeit a matter of conjecture, this could still suggest that rye was used in brewing in the Middle Ages [19]. Nowadays, rye is mainly used in the beverage industry for beer [20] and whisky production [21]. In brewing, it mainly serves as an adjunct, that is to say, a source of fermentable extract at a reduced cost [22]. It is the key ingredient of traditional drinks such as kvass, a Russian fermented beverage made of rye bread [23], and historical beers including the Bavarian Roggenbier and the Finnish sahti, a farmhouse ale flavored with juniper branches and fermented with baker’s yeast [24]. Moreover, rye enters the grist of beer styles such as Belgian Pale Ale [25], Saison and Specialty India Pale Ale (Rye IPA), sought after for its spicy and pungent aroma as a complementary ingredient to barley malt [26]. The malting of rye is similar to that of wheat, although its germination tends to be faster compared to other seeds [27]. Rye grain shares some features with wheat such as rapid water absorption and the potential to give more extract than barley. However, the grain is more prone to damage due to the lack of husks [28]. 

Information on the malting of rye is limited compared to that of wheat and barley. In this study, the response surface methodology was applied to the micro-malting of rye to investigate how three malting parameters (germination time, germination temperature, and degree of steeping) can influence the quality of derived malts. RSM consists of a set of mathematical and statistical tools to develop and optimize processes in a wide variety of research fields [29]. Appropriate mathematical models are defined to investigate the relationship between process parameters and responses of interest to determine optimum conditions [30]. The final stage of this methodology may consist of the simultaneous optimization of several conflicting objects. Widely used methods include the desirability function and the genetic algorithm. The former was originally proposed by Harrington (1980), and his approach was later modified by Derringer and Suich (1984) using a discontinuous function [31]. Theoretical and practical research on genetic algorithms based on biological evolution has been conducted for over 40 years by John H. Holland and his research team since the mid-1960s [32]. Genetic algorithms can be successfully combined with the desirability function using models derived from response surface analysis [33,34]. A genetic algorithm consists of at least the following three operators: reproduction, crossover and mutation. A population composed of individuals or chromosomes exchanges information through reproduction. Crossover and mutation operators are involved in the creation of the new generations. Thus, the offspring will result from the combination of information from both parents [35]. Selection of the fittest is based on an objective function (fitness function), i.e., a measure of goodness to be increased. Thus, strings with greater values will have a higher likelihood of transferring their information to subsequent generations, similar to the adaptive process of natural selection and survival of the fittest [36]. To the best of our knowledge, this is the first work in which the malting process of rye has been optimized using two different multi-objective optimization techniques.

## 2. Materials and Methods

### 2.1. Raw Material

A landrace of rye (*Pollino*) with a moisture of 11.0% and a protein content of 14.8% d.m. was used in the micro-malting experiments. The raw material was supplied by a local farm located in the Pollino UNESCO Global Geopark in Calabria region (southern Italy). The initial germinative energy of the grain was 90%, therefore, samples of rye were subjected to 40 °C for a week, in a temperature-controlled chamber, in an attempt to increase it. Afterward, the germination energy remained almost identical. For this reason, the rye samples were malted without any preliminary treatment. Grain samples showed great variability in size, being mostly small and tapered. The results of the sieving test, according to EBC method 3.11.1 for barley, were as follows: grade I, 8.7; grade II, 30.1; rejects, 61.2. Before malting, samples were cleaned and sieved using a sieve machine with sorting sieves (SLN Sample Cleaner Pfeuffer). Both fractions I and II (large and small seeds, respectively) were malted. Although it is recommended to use similar-sized grains to achieve the most uniform malting, in this case, smaller and larger grains were malted together as they were representative of the starting material.

### 2.2. Experimental Design

Response surface methodology was used to generate the experimental domain and for data analysis. A face-centered central composite design (FCCD), broadly used in research on micro-malting optimization [37,38,39], was chosen to set up the experiments. This experimental design is shaped in the form of a cube with the axial points located at the center of the cube faces, with α = 1. The malting process parameters were selected as germination time (d), germination temperature (°C) and degree of steeping (g/100 g). The germination time (A) varied from 4 to 6 days, the germination temperature (B) from 12 to 18 °C, the degree of steeping (C) from 40 to 46 g/100 g. The central points were defined at 5 days, 15 °C and 43 g/100 g. Factor levels were converted in coded units ranging from −1 to +1. Malting parameters into coded form were defined as follows: xA for the germination time; xB for the germination temperature; xC for the degree of steeping. The experimental design consisted of 8 corner points, 4 center points and 6 axial or star points (α), for a total of 18 randomized runs (Table 1). The analyzed responses were as follows: extract, Kolbach index, apparent attenuation limit, viscosity measured in Congress wort and isothermal mash. 

### 2.3. Micro-Malting Plan

For each combination of process parameters, 1 kg of rye was malted in the micro-malting plant of the Research Centre Weihenstephan for Brewing and Food Quality (Freising, Germany), Technical University of Munich (TUM). Due to technical issues, the experiment was completed by adopting the same procedure at the malting facility of the Chair of Brewing and Beverage Technology, Technical University of Munich (TUM). The steeping process was conducted as defined as follows: two stages of imbibition in water of 5 and 4 h were followed by air rests of 19 and 20 h, respectively, for a total of 48 h. The steeping temperature was set according to the experimental design. The moisture level for each sample was brought to the three different target levels on the first day of germination according to the experimental design. It was then maintained at the desired level by spraying water on the following days. Germination time was varied according to the experimental scheme. The samples were subjected to gentle rotation throughout the process to avoid packing. Once the germination step was completed, the samples were subject to the following kilning schedule: 50 °C for 16 h, 60 °C for 1 h, 70 °C for 1 h and 80 °C for 5 h. Afterward, dry rootlets were removed and the different malts were stored for one week prior to analysis. All malt samples presented a moisture content of less than 5%.

### 2.4. Chemical Analyses

Malts and worts were analyzed in duplicate (n = 2) according to the methods of the Mitteleuropäische Brautechnische Analysenkommision (Central European Brewing Technology Commission or MEBAK) [40], using Congress and isothermal 65 °C mash programs. The mean of each measurement has been reported. Before malting, some quality attributes of grain were evaluated. The moisture content of the raw material was measured by means of R-110.40.020 method (Drying Oven Method) as difference in mass before and after drying, while the R-110.29.612 method (AUBRY Method) was used to assess the germinative energy. The nitrogen content was determined by R-110.41.030 according to Kjeldahl method. The protein content was derived by multiplying the nitrogen content by the conversion factor of 5.83 [41]. The moisture content of malts was determined by R-200.18.020, as mass loss over a standardized drying procedure. Soluble nitrogen and protein content were measured according to R-205.11.030 and R-200.20.030, respectively, according to Kjeldahl method. Kolbach index was calculated as the ratio of soluble to total nitrogen, according to R-205.12.999 method. The extract was assessed on the Congress mash by R-205.01.080 method. Apparent attenuation limit (AAL) was measured according to R-205.16.080 method (Fermentation Tube Method). Viscosity was measured in Congress wort and isothermal (65 °C) wort according to R-205.10.282 method using a microviscometer from Anton Paar. 

### 2.5. Statistical Analysis

Data analysis was performed in RStudio, a free software environment for statistical computing and graphics, R version 4.1.0 (2021-05-18). Response surface analysis was carried out using ‘rsm’ package [42]. The ‘graphics’ package [43] was used to visualize the response surfaces. Model checking adequacy was conducted by means of ’performance’ [44], and ‘mass’ packages [45]. Data visualization was realized using basic functions of R, ‘ggplot’ [46], ‘viridis’ [47] and ‘patchwork’ [48] packages. Multi-criteria optimization was conducted using ‘desirability’ [31] and ‘GA’ [35] packages. Malting at 6 days, 15 °C and 43 g/100 g (run No. 10) was not possible due to technical issue related to the malting plant. The handling of missing data generally consists of the following two strategies: analyzing an incomplete dataset or resorting to strategies such as multiple imputation. In the latter case, missing data are replaced with new values on the basis of experimental observations [49]. In order to check whether missing values could alter the predicted results and thus influence the goodness of the method, missing values were replaced by means of multivariate imputation by chained equations (data not shown) using the ‘mice’ package [50]. It was possible to conclude that the results with and without imputation were comparable. In light of this, it was preferred to keep the missing values during the data analysis.

## 3. Results and Discussion

### 3.1. Model Fitting and Adequacy Evaluation

The first important step in the response-surface analysis is to set up the relationship between natural and coded variables, as given in the following equations below:

(1) xA=(A−5)/1

(2) xB=(B−15)/3

(3) xC=(C−43)/3

where the value of a coded variable is equal to the difference between the uncoded form and its center points, divided by half the variable’s range. Coding ensures that all input variables are dimensionless and vary over the same interval, so as to guarantee a feasible interpretation of the surface analysis [42]. Quadratic or second-order models were defined to approximate true response functions. The models’ performance was compared on the basis of statistical fit indices and the significance of the models’ coefficients. The Akaike information criterion (AIC) and Bayesian information criterion (BIC) are widely used as indicators to select the most suitable models. In brief, the fitted models with the lowest AIC and BIC and the highest adjusted R squared were selected for data analysis (Table S1). The only exception was the model for the AAL, where only one interaction term was found to be statistically significant. Nevertheless, it was decided to choose the quadratic model, taking into account the relevant values for the adjusted R squared, AIC and BIC, as well as the performance of the model (Table S1).

**Figure 1:**
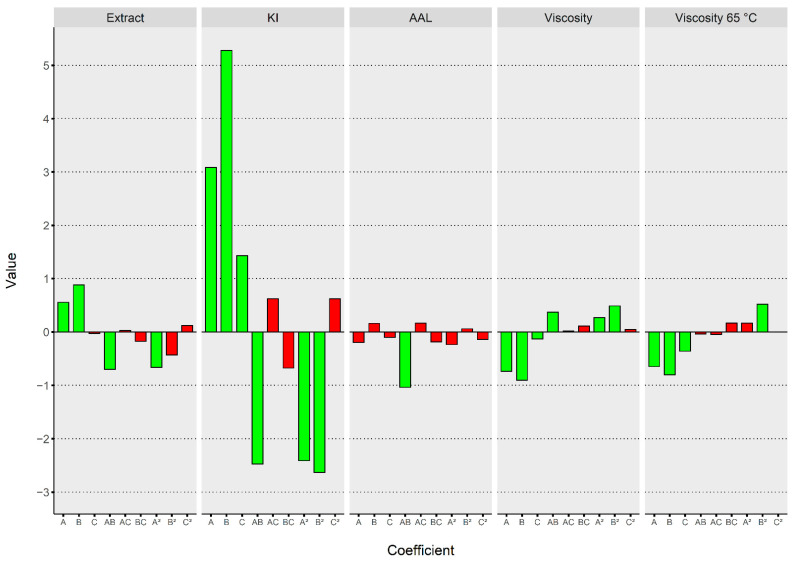
Comparison of coefficient estimates in terms of coded factors. The colors of the bars indicate the significance of each term: green for significant terms, red for non-significant terms. KI, Kolbach index; AAL, apparent attenuation limit; A, germination time; B, germination temperature; C, degree of steeping.

The linear, interaction and quadratic terms were found to be variously significant for the preliminary fitted models (Figure 1). 

At first glance, the process parameters exerted different influences on the responses under study. In particular, the KI variable was the most affected overall, with the germination temperature exerting a large positive impact. All linear terms had a negative impact on viscosities. All models resulted in significance based on the F-values. In developing models within the adopted methodology, attention must be paid to the lack offit test, a statistical indicator used to check the model’s adequacy in predicting response surfaces [51]. Fitted models presented a non-significant lack of fit, so they were considered suitable for making predictions and drawing conclusions from the response surfaces. Table 2 shows the outcomes of the analysis of variance for all selected models.

All ANOVA assumptions were confirmed throughout the analysis of externally studentized residuals and diagnostic plots. Residuals were found to be normally distributed, so the assumption of normality was fulfilled. No particular trend was found for residuals against predicted values (Figure S1) and runs (Figure S2). The points resulting from the combination of predicted and observed values were randomly distributed and close to the fitted lines (Figure S3). This is an indication of good prediction for all analyzed models, as also suggested by the values of the coefficients of determination that resulted close to the unit. The comparison between actual and predicted outcomes is reported in Table 3.

### 3.2. Extract

The extract is a measure of malt yield [28] used to estimate the number of soluble substances in the wort [52]. The extract values of rye and wheat malt are generally higher than those of barley malt [28]. Wang et al. (2018) reported average extract values comprised between 85.0 and 85.9% d.m. for germination times between 3 and 6 days, with no significant differences after three days of germination [53]. The observed values ranged from 80.1 to 83.9% d.m. The minimum extract was obtained after 4 days at 12 °C and 46 g/100 g, while the maximum was obtained after 5 days at 18 °C and 43 g/100 g, respectively. A similar extract (83.8% d.m.) was also reached after 5 days, at a lower temperature and a higher degree of steeping (15 °C and 46 g/100 g, respectively). Values beyond 83.0% d.m. could be obtained at every level of germination time and degree of steeping at temperatures ranging between 15 °C and 18 °C. The quantity of extract measured after 6 days of germination showed a different trend depending on the temperature degree of the steeping combination, according to an inversely proportional relationship. Therefore, by raising the temperature from 12 to 18 °C with a simultaneous reduction of the degree of steeping from 46 to 40 g/100 g, an increase in the extract from 83 to 83.5% was observed. Indeed, it is well known that a gradual increase in the moisture content of the germinating seed enhances the embryo’s growth rate and respiration, resulting in reduced extract content [28]. The best-fitted model describing the response was the following:

(4) $$Extract=83.3652+0.5636\times xA+0.8800\times xB-0.7000\times xAxB-0.5929\times xA^{2}-0.4109\times xB^{2}$$

**Figure 2:**
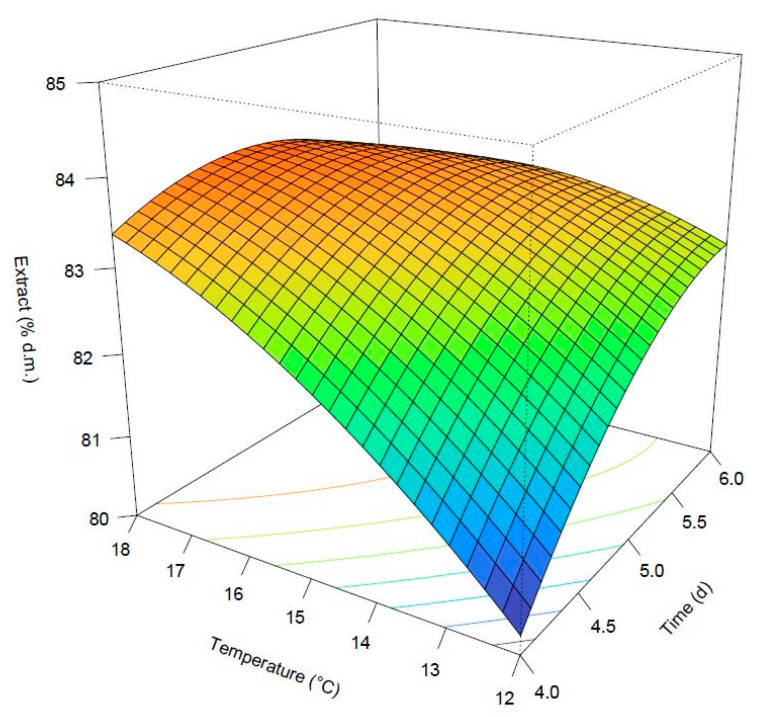
Surface plot of the predicted extract.

The adjusted R squared was high and close to the unit (0.937), and the lack of fit was not significant (*p*-value: 0.9103533). Germination time and temperature exerted a major role in the response. They both presented positive coefficients, which means increasing A or B determined a consequent increase in the predicted response. Nonetheless, their interaction was also significant. Its coefficient presented a negative sign, which means the effect of A on the response will decrease by raising B and vice versa. The quadratic coefficients for the same parameters were also significant. The degree of steeping did not exert any significant influence on the extract, and for this reason, it was not considered in the model. Figure 2 shows the 3D surface of the predicted response based on the fitted model. It was possible to identify a point near-maximum at about 5 days at 18 °C. Predicted extracts equal to 83.7% could be obtained for temperatures comprised between about 17 and 18 °C and for germination times ranging from 4.5 to 5.5 days. 

### 3.3. Kolbach Index

KI is defined as the ratio of soluble to total nitrogen and indicates the degree to which the malt is modified [40]. It is a measure of the proteolytic activity that occurs in malting. The recommended range for barley malt for short-infusion mashing is 38–42% [54]. The highest soluble nitrogen contents were found at higher temperatures for long germination times (data not shown), as also reported in Hübner & Arendt (2010) [55]. The minimum observed value (43.3%) was attained at 4 days, 12 °C and 40 g/100 g, while the maximum (62.0%) was attained at 5 days, 15 °C and 46 g/100 g. Overall, lower values of KI have been detected if compared with the results reported in Wang et al. (2018) [53]. These differences can presumably be attributed to the different experimental designs used as well as genotypic differences. Increasing the germination time from the lowest to the highest level while keeping the temperature and degree of steeping at a constant level of 12 °C and 40 g/100 g, respectively, determined an increase in KI from 43.3 to 51.8%. A quadratic model described the response as follows:

(5) $$KI=59.3654+3.0837\times xA+5.2800\times xB+1.4300\times xC-2.4750\times xAxB+0.6250\times xAxC\;-0.6750\times xBxC-2.4125\times xA^{2}-2.6308\times xB^{2}+0.6192\times xC^{2}$$

**Figure 3:**
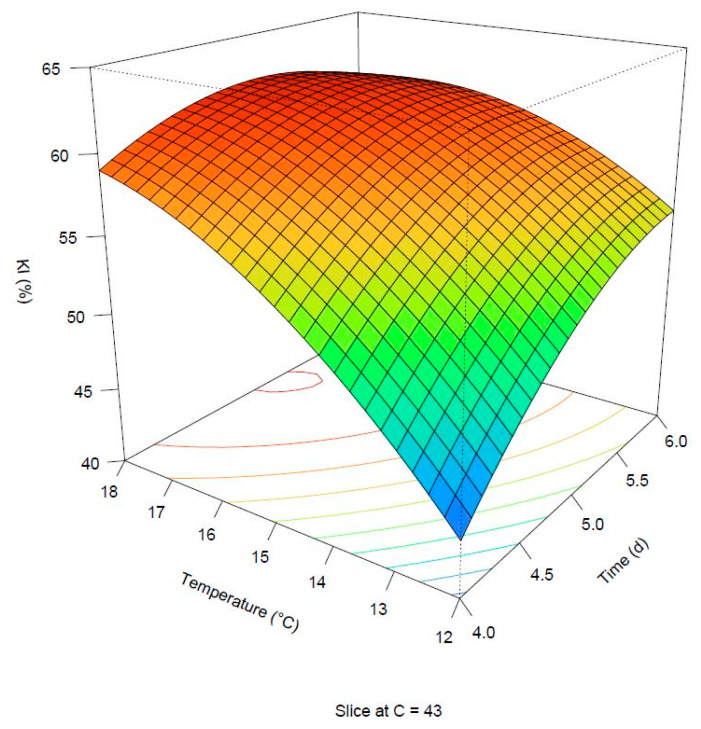
Surface plot of the predicted Kolbach index. The degree of steeping is held at 43 g/100 g.

The adjusted R squared was equal to 0.946, which means most of the variability observed was explained by the selected model. The lack of fit was not significant (*p*-value: 0.128669). All the linear terms were significant, with the germination temperature exerting the strongest influence on the response (Figure 1). The interaction term between germination time and the temperature was also significant, and the coefficient presented a negative sign. They both contributed to influencing the KI response; for this reason, the effect of A on KI resulted less strong with a concurrent increase in B and vice versa. There was a discrepancy regarding the combination of experimental settings allowing to reach the maximum response if observed and predicted values were compared. In fact, the maximum KI (62.2%) was predicted at 6 days, 18 °C and 46 g/100 g. Nonetheless, an area of maximum predicted response (>62.0%) has been predicted by the fitted model in the range 5–5.5 days and 17–18 °C when the degree of steeping was held at a constant level of 43 g/100 g, as depicted in Figure 3.

### 3.4. Apparent Attenuation Limit

The apparent attenuation limit measured in the Congress wort represents the difference in extract before and after fermentation [40]. In particular, the term attenuation refers to the decrease in the specific gravity of the wort and thus its sugar content after fermentation [28]. The recommended value for barley malt is over 80% [56]. The observed responses were in the range of 71.5–74.7%. The minimum value was reached at 6 days, 18 °C and 46 g/100 g, while the maximum value was reached at 4 days, 18 °C and 40 g/100 g. An average AAL of 73.0% was obtained at the central points of the experimental region. The second-order model describing the AAL response is shown as follows:

(6) $$AAL=73.0460-0.1990\times xA+0.1600\times xB-0.1000\times xC-1.0375\times xAxB+0.1625\times xAxC\;-0.1875\times xBxC-0.2375\times xA^{2}+0.0577\times xB^{2}-0.1423\times xC^{2}$$

**Figure 4:**
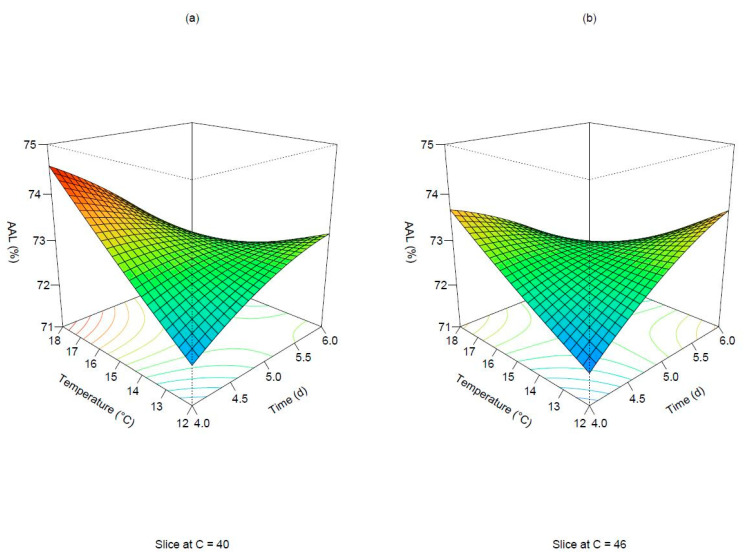
Surface plots of the predicted apparent attenuation limit. The degree of steeping is held at 40 g/100 g (**a**) and 46 g/100 g (**b**).

The adjusted R squared was the lowest (0.909) among the analyzed responses. The lack of fit was not significant (*p*-value: 0.1212). No parameters exerted a statistically significant influence when considered individually. The only regression coefficient resulting in significance was the interaction effect of germination time and temperature. There was correspondence between the observed and predicted minimum and maximum values for the same combination of parameters. The resulting response surfaces can be defined as a saddle point or min-max, thus an optimum area could not be identified. Figure 4 illustrates the pattern of the response as time and temperature change when the degree of steeping is kept constant at 40 g/100 g and 46 g/100 g. Three different situations were identified by setting the degree of steeping at the three different levels defined by the experimental design. AAL values above 74.0% could be detected for short germination times and high temperatures when the degree of steeping was held at 40 g/100 g (Figure 4, subplot a). In particular, as the degree of steeping increased from the lowest to the highest level, the response surface tended to shift within lower boundaries defined by the predicted AAL. Areas of increasing response can be observed both at 4 days and 18 degrees and at 6 days and 12 degrees when humidity is maintained at the highest level (Figure 4, subplot b). Generally, the minimum response tended to be located at the corner of the experimental region, at the lowest and highest combination levels for germination time and temperature.

### 3.5. Viscosity

Viscosity can be measured in both Congress and isothermal (65 °C) wort. Both methods provide information on the cytolysis modification of the malt. In particular, the latter gives an insight into the differences between different malts [40]. Recommended values for barley malt are below 1.56 and 1.60 mPa ×s for viscosity measured in Congress and isothermal wort, respectively [54]. Highly modified malts exhibit lower viscosity levels [57]. Unconventional malts other than barley may lead to lautering problems due to the increased wort viscosity [58]. In order to ensure its reduction to levels that allow for good filtration, enzyme activity must be promoted by appropriate timing and temperatures during brewing. Indeed, as amylolytic enzyme efficiency decreases as a result of an excessive shift from their temperature optimum, viscosity tends to rise [59]. The lowest viscosity levels were obtained in malts with extracts close to or above 83%. The ranges of observed values were slightly higher for the viscosity measured in the isothermal mash (5.42–8.93 mPa × s) than in the Congress mash (from 4.19 to 7.89 mPa × s). Both responses were described by a quadratic model, and their equations are reported as follows:

(7) $$Viscosity=4.7654-0.7291\times xA-0.9050\times xB-0.1350\times xC+0.3675\times xAxB+0.0150\times xAxC\;+0.1100\times xBxC+0.2929\times xA^{2}+0.4933\times xB^{2}$$

(8) $$Viscosity\;65\;{}^{\circ}C=6.4582-0.6624\times xA-0.8020\times xB-0.3590\times xC-0.0375\times xAxB-0.0500\times xAxC\;+0.1675\times xBxC+0.6258\times xB^{2}$$

**Figure 5:**
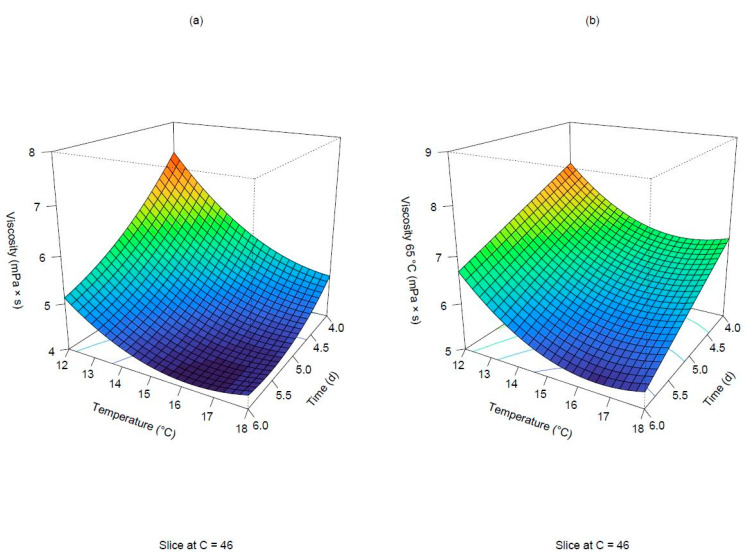
Surface plots of viscosities. Predicted viscosities in Congress (**a**) and isothermal mash (**b**) when the degree of steeping is held at 46 g/100 g.

The adjusted R squared for both models was higher than 0.93, and the lack of fit was non-significant (*p*-values: 0.0825167 and 0.2749261 for viscosity measured in Congress and isothermal wort). All linear terms, the interaction and the quadratic terms for germination time and temperature resulted in significance for the viscosity measured in the Congress wort. In particular, both factors exerted a greater negative influence. A negative effect was also exerted by the degree of steeping, but to a lesser extent. In both scenarios, it can be concluded that as time, temperature and degree of steeping increase, a consequent drop in viscosity is expected. Under these conditions, a gradual cytolytic modification of the cell wall components affecting the wort viscosity occurs. Figure 5 shows the interactive effect of time and temperature when the degree of steeping is held at a constant level of 46 g/100 g for both predicted responses. As depicted in the two subplots, the response surfaces turned into a concave shape. Prolonging the germination time at increasing temperatures resulted in a decrease in the predicted responses. Particularly, a plateau can be observed below 15 °C and after 5 days of germination, at which the lowest viscosity values were predicted by the two models.

### 3.6. Multicriteria Optimization

The optimization process was performed by means of the following two different methods: the desirability function approach and the genetic algorithm. The target was to maximize extract and ALL, while keeping the KIand the viscosity measured in Congress and the isothermal wort as low as possible. Therefore, the function was set up in view of the validity of the fitted models and in such a way that all the following three events occurring during malting are accounted for: amylolysis, proteolysis and cytolysis. KI was considered an indicator of proteolysis. A balance between soluble nitrogen and protein content is generally sought, so a KI within a certain range is preferable. High values may adversely affect certain beer characteristics. For instance, foam stability in beer was found to be negatively correlated to KI, although with different magnitudes [60,61]. Overall, the measured KI values were remarkably high (43.3–62.0%) compared to the reference values of barley and wheat malt. Taking this into account, when setting the individual desirability function for KI, the aim was to minimize it.

#### 3.6.1. Desirability Function

**Figure 6:**
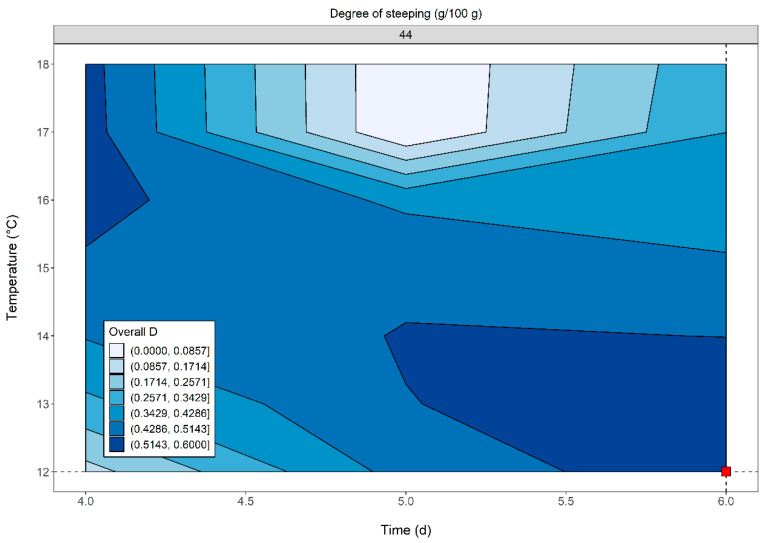
Contour plot of the overall desirability D. Color shades refer to specific D ranges: from light to dark blue, D increases. The dotted lines meet at the highest computed overall desirability value D (0.568), denoted by the red square.

The acceptability boundaries for the responses were set on the basis of the minimum and maximum values of the experimental results. Each response was transformed into an individual desirability value ranging from 0 to 1. A larger-is-better function was applied to maximize extract and AAL. A smaller-is-better function was used to minimize KI and viscosity measured in Congress and isothermal wort. The scaling factor for both maximization and minimization was set at 1. A data frame containing combinations of parameters in coded and uncoded units was created. Based on the fitted models, the selected responses to be optimized were predicted for each combination of independent variables. Hence, individual desirability values were calculated and finally combined to obtain the overall desirability D. Two areas of feasible solutions were identified, as depicted in Figure 6.

Indeed, maximum desirability values were obtained for the same degree of steeping but at opposite levels for the temperature and time parameters. Thus, at a fixed degree of steeping of 44%, satisfactory responses meeting the above criteria can be obtained by combining long germination times at low temperatures, or alternatively for shorter germination periods at higher temperatures. The highest value computed by the function was 0.568 at 6 days, 12 °C and 44 g/100 g. The following optimized responses were predicted for this combination of parameters: extract 82.7% d.m.; KI 55.6%; AAL 73.6%; viscosity 5.27 mPa × s; viscosity (65 °C) 7.07 mPa × s.

#### 3.6.2. Genetic Algorithm

**Figure 7:**
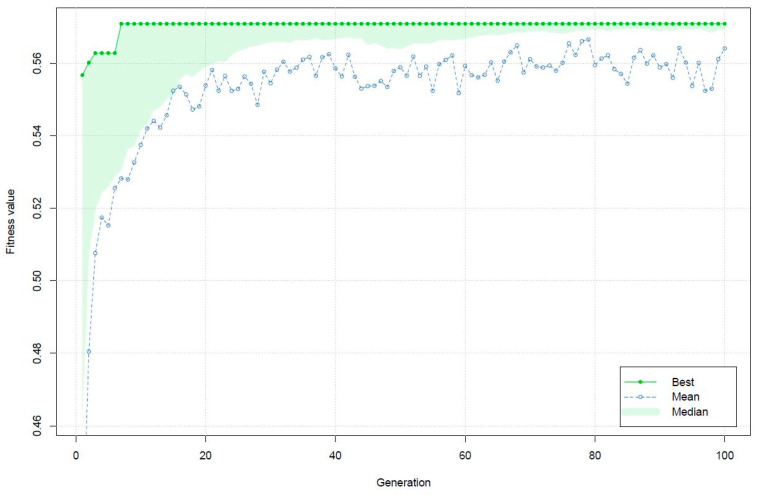
Plot of best, mean and median fitness values recorded during the iterations of the genetic algorithm. The fitness value represents the overall desirability.

A genetic algorithm was applied to find a global optimum implementing the desirability function specified above as the fitness function. Specific information on the definition and typologies of genetic operators can be found in the ‘GA’ package [35]. In the case of optimization problems, the type of genetic algorithm is defined as ‘real-valued’; the minimum and maximum limits of the search space were defined within the boundaries of the experimental design; the population function was defined as ‘gareal_population’, which generates a random and uniform population of real values within the constraints of the search space; selection was defined as ‘gareal_lsSelection’; the genetic operator crossover was chosen as ‘gareal_laCrossover’ which stands for local arithmetic crossover; mutation was defined as “gareal_raMutation” which stands for random and uniform mutation; the elitism operator, equal to the number of individuals with the best fitness surviving each generation or iteration, was set to 5%; the number of individuals within the population was set to 100; the maximum number of iterations was equal to 100; the probability of crossover was set at 0.8; the probability of a mutation occurring within a parent chromosome was set at 0.1; a specific seed was set to generate reproducible results. Each chromosome consisted of combinations of the three independent variables. For each specific combination of parameters within a chromosome, a predicted response was obtained, the fit of which was assessed by means of the fitness function. The worst solution had an overall desirability of 0.4081. After the 7th iteration, the best solution of 0.5709 was identified. Thereafter, no improvement was detected during subsequent iterations (Figure 7).

The best solution found by the genetic algorithm was given in coded units (xA = 1, xB = −0.8352368, xC = 0.06473789). For this reason, the equations defining the relationship between coded and uncoded parameters (Equations (1)–(3)) were used to calculate the real values. The combination of parameters optimizing the overall D was the following: 6 d, 12.5 °C and 43.2 g/100 g. The regression equations in uncoded units for all models were used to compute the predicted values, taking into account the best combination of predicted parameters as follows: extract 82.9%; KI 56.0%; AAL 73.4%; viscosity 5.11 mPa × s; viscosity (65 °C) 6.89 mPa × s. These results coincided with those predicted by the desirability function for the same parameter combination, however, for a lower total desirability value.

## 4. Conclusions

The adopted experimental design made it possible to discern how malting parameters and their levels can influence the quality characteristics of rye malt. The entire response surface analysis and subsequent optimization process were carried out on Rstudio, the R-integrated development environment, showing how the use of open-source software can prove effective as an alternative to expensive software. Analysis of the response surfaces revealed that germination time and temperature strongly influenced the modification of rye malt in terms of amylolytic, proteolytic and cytolytic activity. The influence of the degree of steeping, on the whole, was less pronounced. The desirability function and the genetic algorithm gave similar results. The first method allowed us to navigate a broader space of possible combinations. Nevertheless, the overall desirability D calculated by the genetic algorithm was slightly higher. Both optimization techniques provided comparable results. Hence, the combination of the two optimization techniques proved to be appropriate as part of the surface response methodology. It is interesting to note that two optimal situations can potentially be used to achieve the desired goals at the same degree of steeping of 44 g/100 g. Malting for only 4 days of germination at high temperatures would significantly reduce the costs associated with the process. Nevertheless, the combination predicted at 6 days and 12 °C would reduce the KI to a greater extent. The cereal under study was characterized by a high protein content, so aiming for the best situation with regard to proteolytic activity was one of the objectives. For this reason, the suggested malting parameter combination for this rye landrace is as follows: 6 days at 12 °C and 44 g/100 g. Further studies would be needed to fully confirm these results and set up new tests by exploring a different experimental region on the basis of the developed models. Nonetheless, the study helped to give a greater understanding of the amylolytic, cytolytic and proteolytic modifications of rye during malting, the information of which is significantly narrower when compared to that of barley and wheat for malting purposes.

## Figures and Tables

**Table 1:** Face-centered design with coded and natural variables.

Run Order	xA	xB	xC	A	B	C
1	−1	1	1	4	18	46
2	0	0	0	5	15	43
3	−1	1	−1	4	18	40
4	0	0	−1	5	15	40
5	1	1	−1	6	18	40
6	0	0	0	5	15	43
7	0	0	0	5	15	43
8	−1	0	0	4	15	43
9	1	−1	−1	6	12	40
10	1	0	0	6	15	43
11	0	1	0	5	18	43
12	1	1	1	6	18	46
13	0	−1	0	5	12	43
14	0	0	1	5	15	46
15	−1	−1	−1	4	12	40
16	−1	−1	1	4	12	46
17	0	0	0	5	15	43
18	1	−1	1	6	12	46

**Table 2:** Analysis of Variance (ANOVA) of the fitted models.

Response	Source of Variation	Df ^1^	Sum Sq ^2^	Mean Sq ^3^	F Value	*p*-Value
Extract	Model	5	18.05	10.9843	48.31	***
	Residuals	11	0.8220	0.0747		
	Lack of fit	2	0.0170	0.0085	0.0949	ns
	Pure error	9	0.8050	0.0894		
KI ^4^	Model	9	513.82	171.275	32.31	***
	Residuals	7	12.37	1.767		
	Lack of fit	4	10.55	2.637	4.3465	ns
	Pure error	3	1.82	0.607		
AAL ^5^	Model	9	10.20	3.39975	18.65	***
	Residuals	7	0.4254	0.06078		
	Lack of fit	4	0.3654	0.09136	4.5680	ns
	Pure error	3	0.0600	0.02000		
Viscosity	Model	8	16.2291	5.7671	106.7	***
	Residuals	8	0.1521	0.0190		
	Lack of fit	5	0.1386	0.0277	6.172	ns
	Pure error	3	0.0135	0.0045		
Viscosity 65 °C	Model	7	13.0247	5.4064	34.32	***
	Residuals	9	0.4879	0.0542		
	Lack of fit	6	0.3979	0.0663	2.2107	ns
	Pure error	3	0.0900	0.0300		

^1^ Df, degree of freedom; ^2^ Sum Sq, sum of squares; ^3^ Mean sq, Mean Square; ^4^ KI, Kolbach index; ^5^ AAL, apparent attenuation limit. The symbols ***, ns, indicate the following levels of significance for each ANOVA model: 0.001, not significant.

**Table 3:** Comparison between actual and predicted responses using RSM.

Run Order	Extract (%, d.m.^1^)		KI ^2^ (%)		AAL ^3^ (%)		Viscosity (mPa × s)		Viscosity 65 °C (mPa × s)	
	Actual	Predicted	Actual	Predicted	Actual	Predicted	Actual	Predicted	Actual	Predicted
1	83.2	83.4	60.6	59.7	73.5	73.7	4.99	4.97	6.93	6.84
2	83.4	83.4	60.3	59.4	73.0	73.0	4.67	4.77	6.36	6.46
3	83.5	83.4	59.6	59.5	74.7	74.6	4.92	5.05	7.13	7.12
4	83.3	83.4	57.3	58.6	72.7	73.0	5.04	4.90	6.88	6.82
5	83.5	83.1	60.6	59.4	71.8	71.8	4.29	4.30	5.99	5.82
6	83.3	83.4	60.4	59.4	72.9	73.0	4.69	4.77	6.12	6.46
7	83.1	83.4	59.3	59.4	72.9	73.0	4.71	4.77	6.54	6.46
8	82.3	82.2	53.2	53.9	73.1	73.0	5.82	5.79	7.42	7.12
9	82.5	82.7	51.8	52.5	73.3	73.2	5.56	5.59	7.77	7.84
10	na ^4^	na ^4^	na ^4^	na ^4^	na ^4^	na ^4^	na ^4^	na ^4^	na ^4^	na ^4^
11	83.9	83.8	60.4	62.0	73.3	73.3	4.55	4.35	5.94	6.28
12	82.7	83.1	61.7	62.2	71.5	71.5	4.19	4.28	5.42	5.34
13	82.1	82.1	52.4	51.5	73.0	72.9	6.00	6.16	8.14	7.89
14	83.8	83.4	62.0	61.4	73.2	72.8	4.63	4.63	6.17	6.10
15	80.3	80.2	43.3	42.6	71.8	71.8	7.89	7.81	8.93	8.99
16	80.1	80.2	44.6	45.6	71.6	71.7	7.29	7.29	7.89	8.03
17	83.2	83.4	58.8	59.4	73.2	73.0	4.82	4.77	6.38	6.46
18	83.0	82.7	58.0	57.9	73.5	73.7	5.25	5.13	6.70	6.68

^1^ d.m., dry matter; ^2^ KI, Kolbach index; ^3^ AAL, apparent attenuation limit; ^4^ na, not available.

## Data Availability

The data presented in this study are available upon request to the corresponding author.

## Supplementary Materials

The following supporting information can be downloaded at: https://www.mdpi.com/article/10.3390/foods11223561/s1, Table S1. Comparison of model performance indices; Figure S1. Plot of externally studentized residuals vs. predicted responses. Each subplot refers to a specific quality attribute: (**a**) extract; (**b**) KI, Kolbach index; (**c**) AAL, apparent attenuation limit; (**d**) viscosity in the Congress wort; (**e**) viscosity in the isothermal mash; Figure S2. Plot of externally studentized residuals vs. run number. Each subplot refers to a specific quality attribute: (**a**) extract; (**b**) KI, Kolbach index; (**c**) AAL, apparent attenuation limit; (**d**) viscosity in the Congress wort; (**e**) viscosity in the isothermal mash; Figure S3. Plot of predicted vs. actual responses. Each subplot refers to a specific quality attribute: (**a**) extract; (**b**) KI, Kolbach index; (**c**) AAL, apparent attenuation limit; (**d**) viscosity in the Congress wort; (**e**) viscosity in the isothermal mash.
