# Supplementary material for: Multi-Response Optimization of the Malting Process of an Italian Landrace of Rye (Secale cereale L.) Using Response Surface Methodology and Desirability Function Coupled with Genetic Algorithm
Source: Foods. 2022 Nov 9;11(22):3561. doi: 10.3390/foods11223561 (PMC9689978; doi:10.3390/foods11223561)
Supplement: Supplementary file 1 [file foods-11-03561-s001.zip › foods-1975894-supplementary.pdf]

Table S1. Comparison of model performance indices.

Response	Model	AIC [1]	BIC [2]	R squared	Adjusted R squared	RMSE [3]	Sigma	Performance score
Extract	FO [6]	45.923	50.089	0.564	0.463	0.696	0.796	0.00%
	TWI [7]	39.922	46.588	0.785	0.655	0.489	0.638	27.48%
	PQ [8]	11.246	20.411	0.972	0.936	0.176	0.275	82.04%
	**CM [9]**	**10.747**	**16.579**	**0.956**	**0.937**	**0.220**	**0.273**	**97.97%**
KI [4]	FO	94.356	98.522	0.730	0.667	2.255	3.308	0.00000122%
	TWI	91.893	98.559	0.836	0.737	2.255	2.940	19.63%
	**PQ**	**64.836**	**74.001**	**0.976**	**0.946**	**0.853**	**1.329**	**97.50%**
	CM	67.011	73.676	0.962	0.939	1.085	1.414	84.93%
AAL [5]	FO	49.119	53.285	0.065	-0.151	0.765	0.874	0.00%
	TWI	13.014	19.679	0.921	0.874	0.222	0.289	67.41%
	**PQ**	**7.551**	**16.716**	**0.960**	**0.908**	**0.158**	**0.247**	**100.00%**
	CM	13.014	19.679	0.921	0.874	0.222	0.289	67.41%
Viscosity	FO	31.257	35.423	0.788	0.739	0.452	0.517	0.0000000289%
	TWI	30.213	36.878	0.860	0.776	0.368	0.479	14.02%
	PQ	-10.507	-1.342	0.991	0.979	0.093	0.145	79.75%
	**CM**	**-11.937**	**-3.605**	**0.991**	**0.981**	**0.095**	**0.138**	**99.90%**
Viscosity 65 °C	FO	24.538	28.705	0.827	0.787	0.371	0.424	5.49%
	TWI	28.572	35.238	0.846	0.753	0.350	0.457	3.86%
	PQ	7.522	16.687	0.969	0.928	0.158	0.246	75.49%
	**CM**	**5.880**	**13.379**	**0.964**	**0.936**	**0.169**	**0.233**	**98.56%**

[1] AIC, Akaike information criterion; [2] BIC, Bayesian information criterion; [3] RMSE, Root Mean Square Error; [4] KI, Kolbach index; [5] AAL, apparent attenuation limit; [6] FO, first order; [7] TWI, two-way interaction; [8] PQ, pure quadratic; [9] CM, customized model (excluding some non-significant terms while maintaining hierarchy) The selected models have been reported in bold.

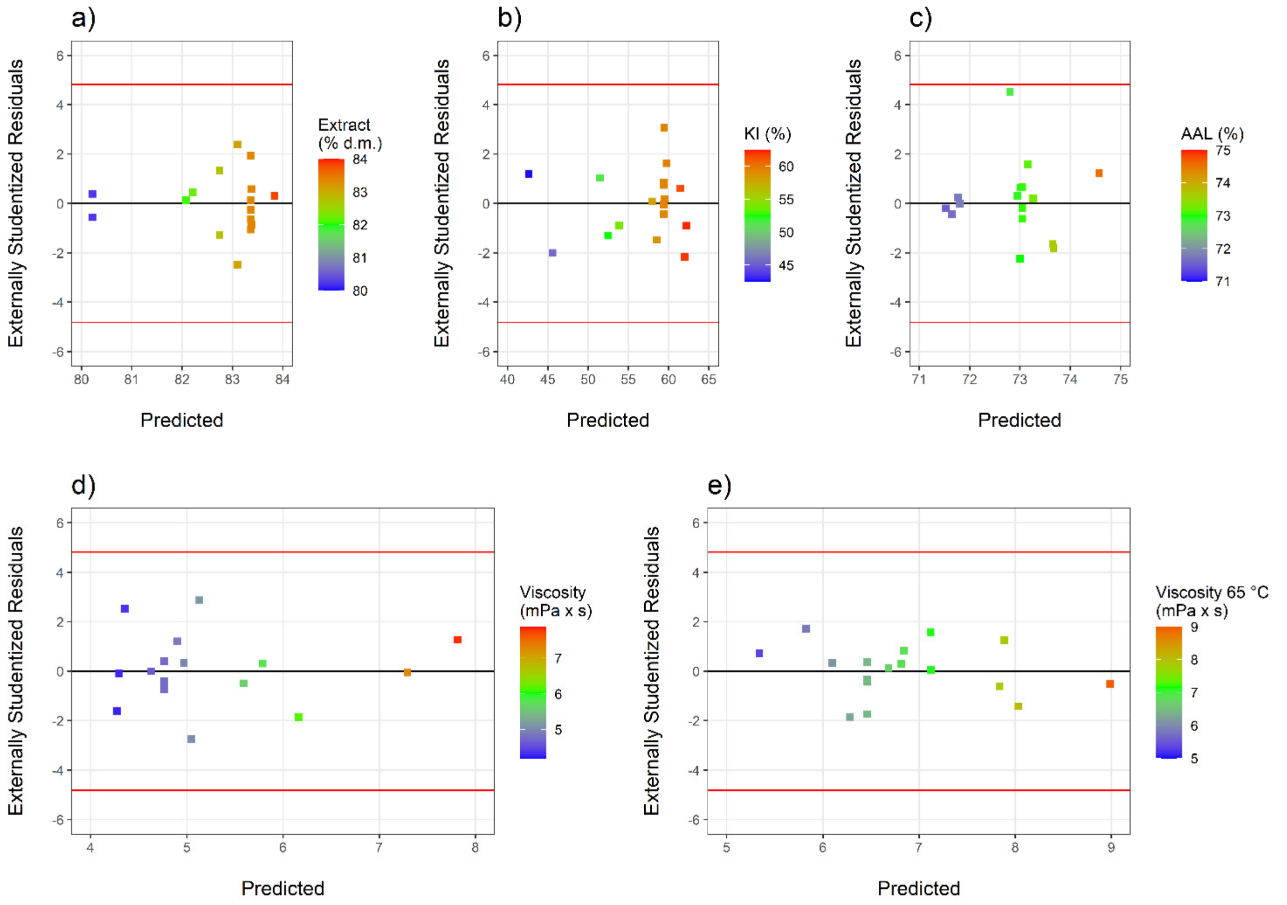

Figure S1. Plot of externally studentized residuals vs predicted responses. Each subplot refers to a specific quality attribute: a) extract; b) KI, Kolbach index; c) AAL, apparent attenuation limit; d) viscosity in the Congress wort; e) viscosity in the isothermal mash.

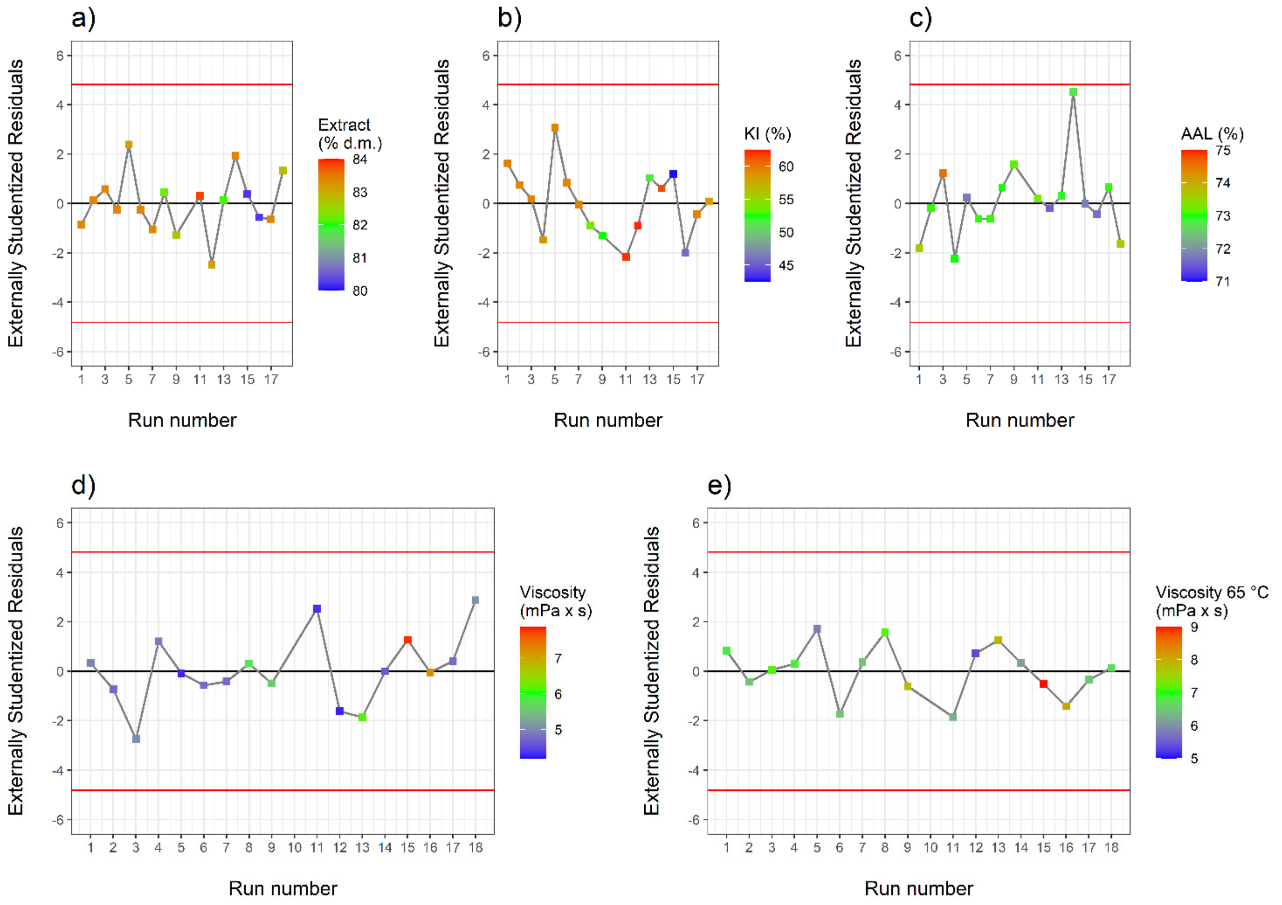

Figure S2. Plot of externally studentized residuals vs run number. Each subplot refers to a specific quality attribute: a) extract; b) KI, Kolbach index; c) AAL, apparent attenuation limit; d) viscosity in the Congress wort; e) viscosity in the isothermal mash.

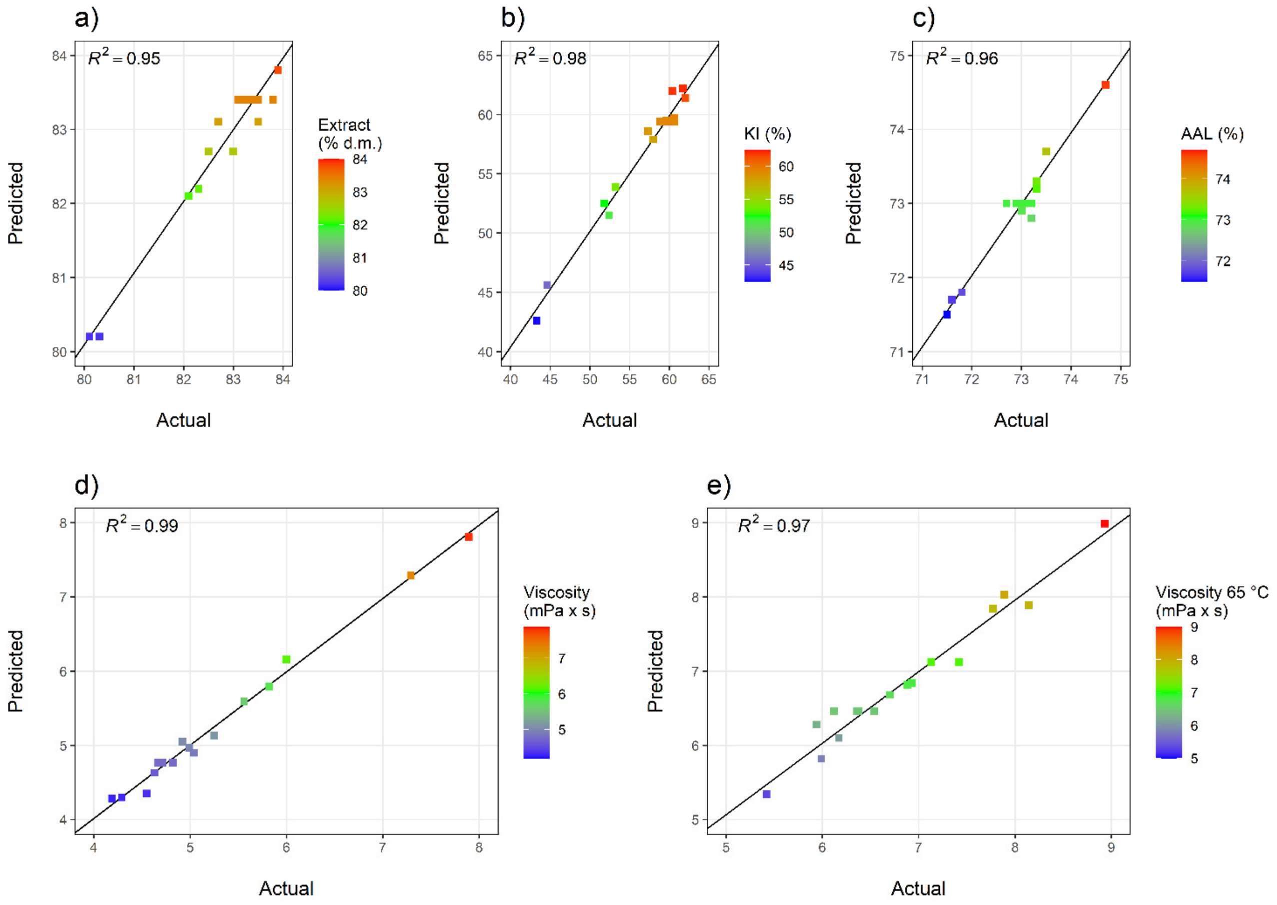

Figure S3. Plot of predicted vs actual responses. Each subplot refers to a specific quality attribute: a) extract; b) KI, Kolbach index; c) AAL, apparent attenuation limit; d) viscosity in the Congress wort; e) viscosity in the isothermal mash.
